# Subthalamic Nucleus (STN)-Deep Brain Stimulation Reduces the Power of Mu and Beta Rhythms and Enhances Synchrony at the Motor Cortices in Parkinson's Disease: A Report of Two Cases

**DOI:** 10.7759/cureus.35057

**Published:** 2023-02-16

**Authors:** Zaitun Zakaria, Zamzuri Idris, Sanihah Abdul Halim, Abdul Rahman Izaini Ghani, Jafri M Abdullah

**Affiliations:** 1 Department of Neurosciences, School of Medical Sciences, Universiti Sains Malaysia (USM), Kota Bharu, MYS; 2 Department of Neurosciences, School of Medical Sciences, Hospital Universiti Sains Malaysia (HUSM), Kota Bharu, MYS; 3 Department of Medicine, School of Medical Sciences, Universiti Sains Malaysia (USM) Kubang Kerian, Kota Bharu, MYS; 4 Department of Neurosciences, School of Medical Sciences, Universiti Sains Malaysia (USM) Kubang Kerian, Kota Bharu, MYS; 5 Department of Neurosurgery, Universiti Sains Malaysia (USM) Health Campus, Kota Bharu, MYS

**Keywords:** beta wave, mu waves, motor cortex, subthalamic nucleus, parkinson disease, deep brain stimulation

## Abstract

The motor circuit in Parkinson’s disease (PD) involves the basal ganglia, thalamus, motor cortex, and cerebellum. Hence, subthalamic nucleus (STN) or globus pallidus internus deep brain stimulation is commonly used in treating refractory Parkinson’s patients. During the procedure, the local field potential (LPF) is commonly made along the trajectory of the STN. Two cases were assessed, where an electroencephalographic recording at the sensorimotor cortices was also performed with and without stimulation at the optimal STN electrode site. The ‘on’ stimulation state associated with clinical improvement correlated with a marked reduction in the late theta (7.5 Hz), alpha (10.5 Hz) (Mu wave), and beta (20 Hz) wave power. Besides, more synchronized and coherent brainwaves were noted when the stimulation was ‘on’.

## Introduction

Parkinson’s disease (PD) has been associated with the loss of pigmented dopaminergic neurons in the substantia nigra and a deficiency in striatal neurogenesis [1,2]. Many theories were shone on the disease progression. In 2003, Braak et al. hypothesized that alpha-synuclein (asyn) aggregates from the gut, invading the brain from the olfactory epithelium via retrograde axonal transport through the vagus and other limbic structures, causing the disease [3]. This concept is supported by epidemiological evidence of pathological asyn aggregates in the peripheral nervous system (PNS) of PD patients up to 20 years prior to diagnosis [4,5].

Meanwhile, the oscillator theory explains the basic anatomy of the basal ganglia-thalamic-cortical system as many sets of interconnected reentrant oscillators or loops [6-8]. A single loop involves multiple structures, and a structure may contain a multitude of oscillators. This oscillating system posits different patterns of wave propagation, which sweep across the motor cortex to orchestrate the proper sequence of muscle activities. Based on the above, the common anatomical target in deep brain stimulation (DBS) is either at the subthalamic nucleus (STN) or globus pallidus internus (GPi). In PD patients, the administration of high-frequency stimulation has decreased the excessive domination of beta oscillations in the motor system [8-10]. The neuronal activity at the sensorimotor cortices during the STN-DBS surgery potentially be captured using an electroencephalogram (EEG). Herein, this study describes two cases of an EEG recording when the STN stimulation was turned ‘on’ and ‘off’.

## Case presentation

Case 1

A 56-year-old lady was diagnosed with PD in 2009. Her initial presentation was bradykinesia, followed by resting tremor initially over her left hand and then the right hand. Her medical therapy, which started in 2011, was complicated by dyskinesia. The efficacy became unpredictable in 2017. Upon examination, she had mask-like facies and slow monotonous speech. Bilateral limb bradykinesia and rigidity were also observed, which were worse over the right side. Her steps were short with festinating gait. The Mattis Dementia Rating Scale (MDRS) scores were mild cognitive impairment (MCI) during her preoperative DBS assessment. A score of 123 to 137 out of 144 is considered MCI.

A Unified Parkinson’s Disease Rating Scale (UPDRS) was used to assess the on-time and off-time. The scores showed significant improvement, with an on-time score of 11 and an off-time score of 34. In view of recent evidence of improvement of all cardinal features of PD patients with STN stimulation, our center targets STN in all PD patients [11]. Furthermore, we achieved an optimal target and better outcome with DBS-STN surgery. Hence, the patient was offered and consented to the treatment.

Case 2

A 61-year-old lady was presented with bradykinesia in 2012. Later, she developed a resting tremor and worsening rigidity. Her symptoms are similar to the first patient, which worsened despite medical intervention. After seven years, the effect became unpredictable. Upon examination, she had mask-like facies and monotonous speech. Her bradykinesia and rigidity were marked over the right side compared to the left. Her bradykinesia has rendered her wheelchair-bound. In 2019, she was assessed for DBS suitability. Her MDRS score also showed MCI while her UPDRS score during on-time was 22 compared to the off-time of 46. She was consented and subsequently subjected to STN-DBS surgery.

STN-DBS and Motor Strip EEG Procedure

**Figure 1:**
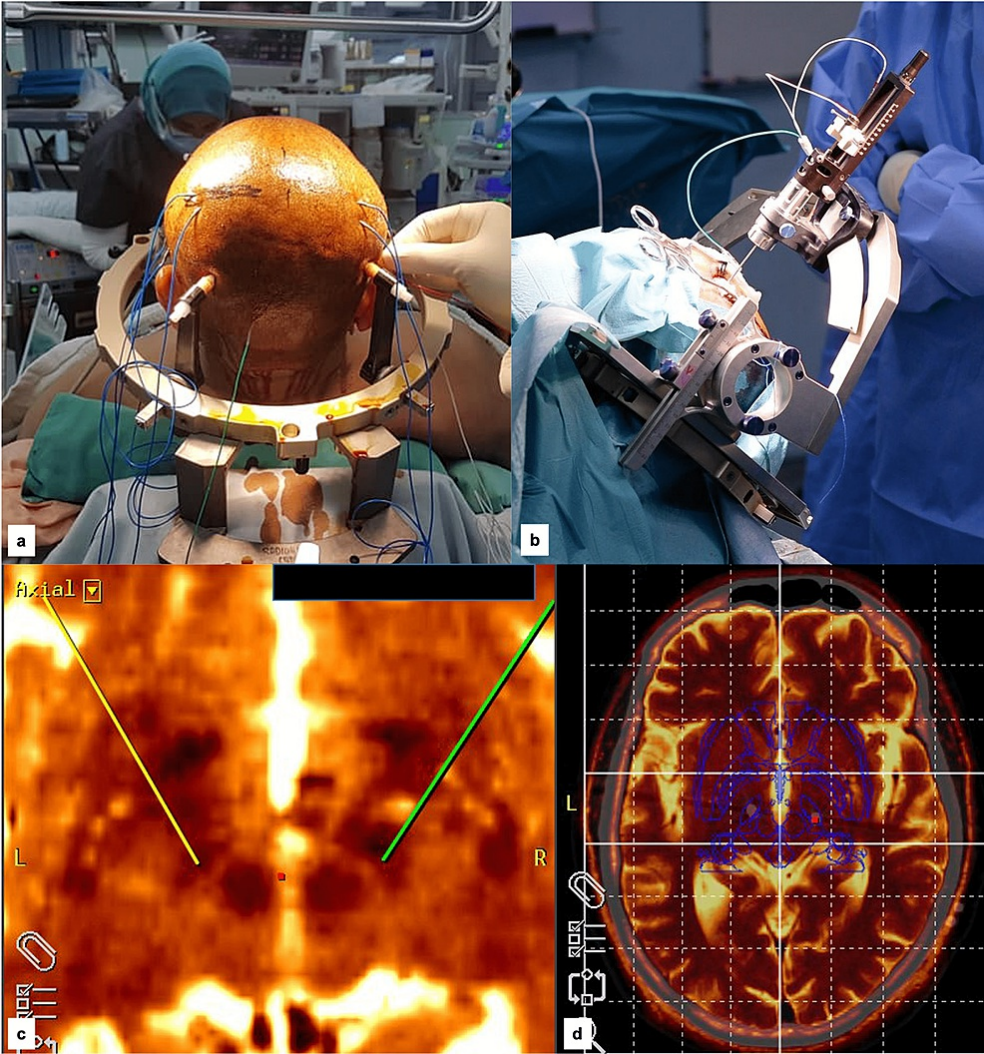
STN-DBS procedure and hand sensorimotor needle scalp EEG (a) Area of the sensorimotor cortex was identified topographically and needle EEGs were inserted deep into the pericranium. (b) STN-DBS procedure using Cosman–Roberts–Wells (CRW) head frame. (c) Bilateral STN targeting and planning based on Framelink. (d) The permanent electrode final site was confirmed with image fusion of the postoperative CT axial image with the preoperative MRI (white and red dot). STN: subthalamic nucleus; DBS: deep brain stimulation; EEG: electroencephalogram

Prior to a standard STN-DBS procedure, under local anesthesia, the brain topographical method was used to allocate the hand sensorimotor strip of the cortex on both hemispheres for needle EEG insertion (Figure 1a: only lateral from midline hand sensorimotor strips were studied). The sensorimotor strip and the central sulcus were identified. The central sulcus was approximated by marking the superior Rolandic point on the scalp (5 cm posterior to the bregma. The inferior Rolandic point lies 4 cm above the preauricular depression (front of the tragus). The connection of these two lines is the estimated central sulcus. Then, DBS surgery proceeded as usual with bilateral STN trajectories planned as shown in Figures 1b-1c using Medtronic StealthStationS7 Framelink software (Minneapolis, Minnesota). The X, Y, and Z coordinates in reference to the AC-PC line for our first patient were 12.0 mm, -1.55 mm, and -5.86 mm on the right side and -12.0 mm, -1.55 mm, and -5.86 mm on the left side, respectively. For the second patient, the values for X, Y, and Z were 13.02 mm, -4.12 mm, and -5.0 mm on the right side and -12.0 mm, -4.0 mm, and -5.0 mm on the left side, respectively. The microelectrode recordings were first made using Medtronic Leadpoint^TM^. After optimal identification of STN recording, macrostimulation to a single electrode was started at 1 mA until the best responses with minimal side effects. In both cases, the best responses were noted at 3 mA of 130 Hz high-frequency stimulation with 60 ms pulse duration. At this moment, the motor cortices scalp EEG with sampling parameters of 512 Hz sampling rate, 70 uV/cm sensitivity, and analyzed frequency from 0 to 30 Hz were simultaneously recorded (NicoletOne^TM^ EEG system; Natus Medical Incorporated, Pleasanton, California), with the sensor-skin impedance maintained below 10 kΩ. The on- and off-stimulation procedure at 3 mA was repeated three times, in which the ‘on’ (clinical improvement noted in the precision of movement, rigidity, and tremor) and ‘off’ (back to previous abnormal limb features). The corresponding parts of EEG during the STN stimulation ‘on’ and ‘off’ state at 3 mA were selected for power spectral density (PSD) analysis when the extremities were at rest. This quantitative EEG (qEEG) software is readily available in the NicoletOne EEG system. Finally, the bilateral permanent macroelectrodes were implanted and connected to the extension wires, tunneled, and connected to an internal pulse generator (IPG) at the right subclavicular region. The postoperative CT brain and preoperative MRI were fused to confirm the site of permanent electrodes (Figure 1d).

Results: Power Spectral Density Analysis

**Figure 2:**
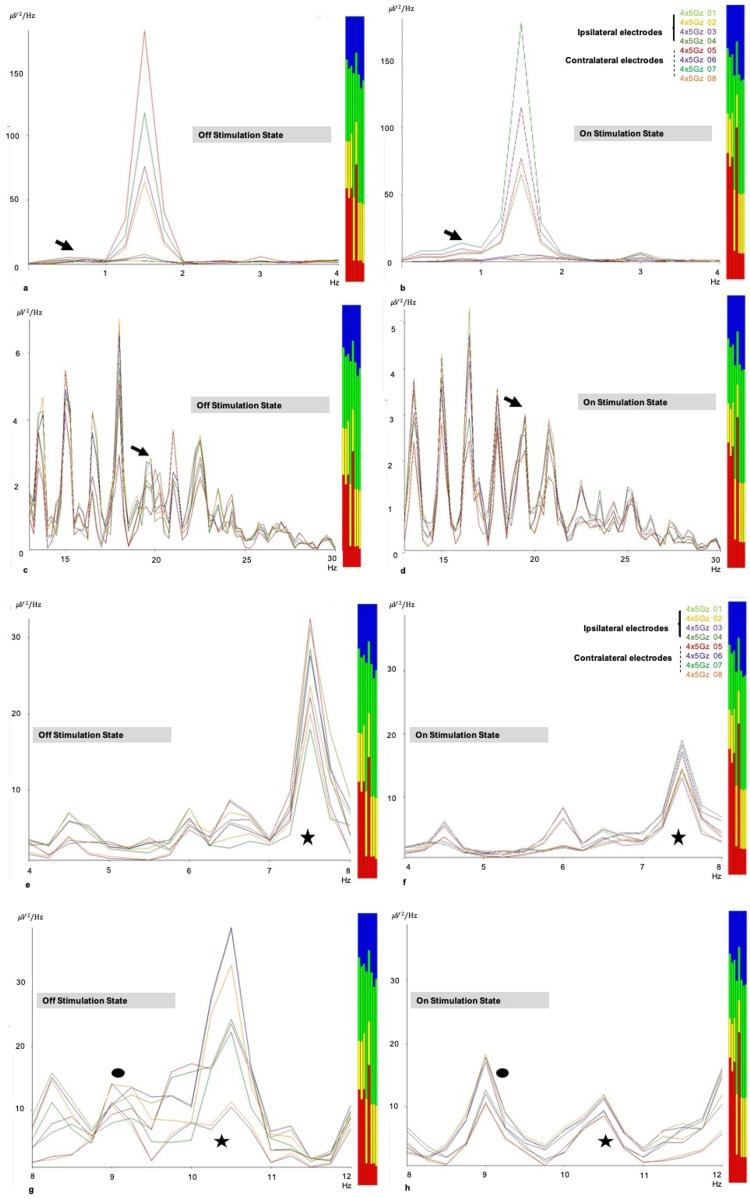
Power spectral density analysis in Case One (a, b) A slight increment in delta wave power at motor cortices was noted when ‘on’ the STN stimulation and the patient showed marked clinical improvement (black arrow). (c, d) Synchrony of the beta waves (particularly at 20 Hz) was observed when ‘on’ the stimulation (black arrow). (e, f) A marked reduction in higher theta (7.5 Hz), and (g, h) alpha (10.5 Hz) wave power was observed when the STN was stimulated and the patient showed clinical improvement (black star). The brainwaves at motor cortices became more synchronized when stimulation was ‘on’ especially noted at the area labeled as a round black dot (at 9 Hz).

PSD analyses for Case One were represented for the ipsi- and contralateral electrode brainwave power. The data were analyzed as a total brainwave power in both hemispheric motor cortices. The analysis revealed a slight change in delta power, with higher values during the on-stimulation (Figures 2a-2b, with the black arrow). Meanwhile, the frequency band around 20 Hz of the beta waves is more synchronized during stimulation, and the patient shows clinical improvement (Figures 2c-2d, with the black arrow). For theta (Figures 2e-2f) and alpha (Figures 2g-2h) power analyses, a marked reduction in brainwave power was noted at 7.5 and 10.5 Hz during stimulation, and the patient showed clinical improvement. It is noteworthy that the brainwaves were more synchronized when the stimulation was ‘on’.

**Figure 3:**
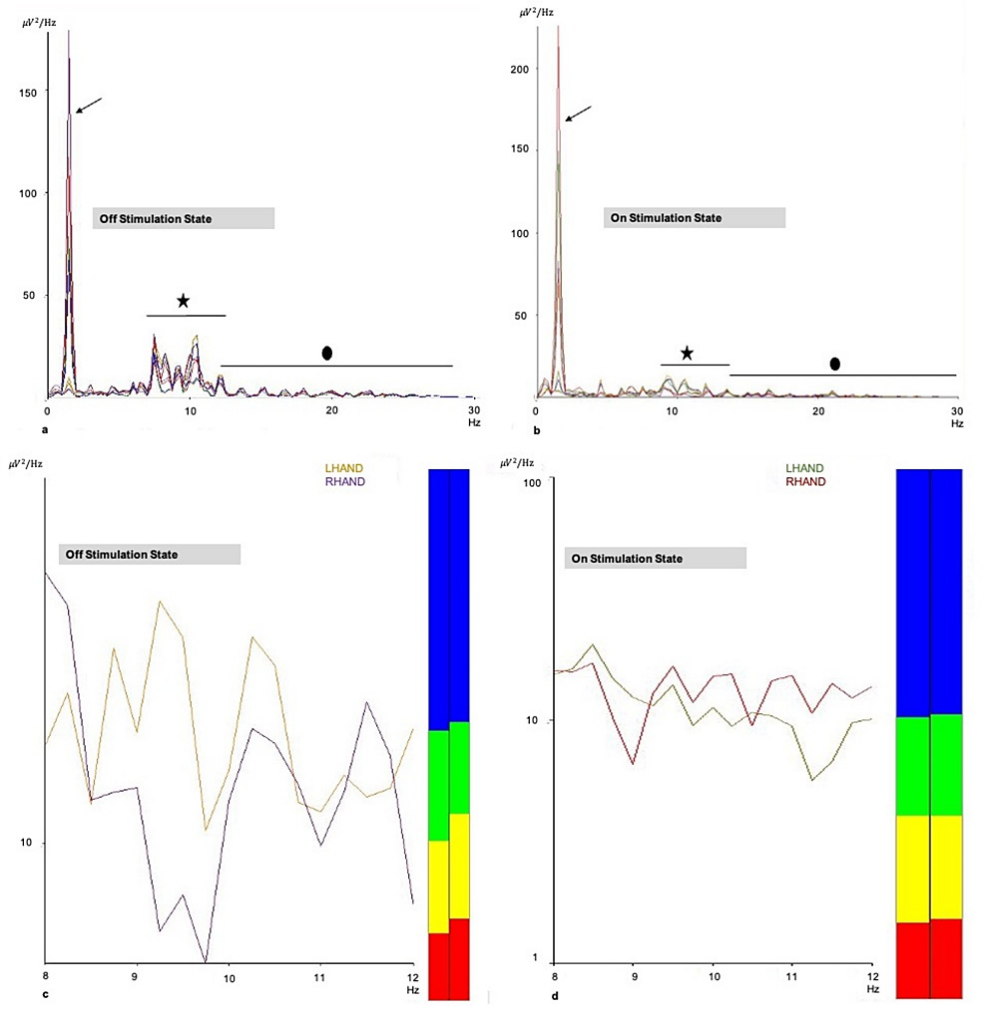
Power spectral density analysis in Case Two (a, b) A slight increment in delta wave power (black arrow) and a marked reduction in Mu and beta wave power were observed when STN was stimulated and the patient showed a good clinical response. (c, d) Synchronized brainwaves were observed in both motor cortices when one of the STN was stimulated and the patient showed a good clinical response on the opposite hand. Blue: beta waves; green: alpha waves; yellow: theta waves, and red: delta waves. LHAND represents an electrode in the opposite motor cortex to the stimulated STN. RHAND represents a motor cortex electrode in the ipsilateral side of the stimulated STN. STN: subthalamic nucleus

Meanwhile, PSD analyses for Case Two are shown in Figures 3a-3b, which re-portray the first initial case finding on brainwave power during the on- and off-stimulation. It reconfirmed the initial case findings where a slight increment in delta wave power and a marked reduction in brainwave power was noted for frequencies ranging from 7 to 11 Hz and 20 Hz (Mu wave (late theta and alpha) and beta wave). The second case also reconfirmed the importance of synchronized brainwaves for the brain to function properly. Figures 3c-3d re-illustrate the finding, where the right and left motor cortices became nearly equalized (enhanced synchrony) in terms of brainwave power for all the waves (colors) and alpha band (lines).

## Discussion

Abnormally excessive oscillation of brainwaves, such as beta oscillation, is present in PD and has been shown to be decreased after the application of dopamine and DBS [12-14]. Consequently, sensorimotor cortex brainwave studies on healthy subjects revealed the importance of theta, alpha, and even beta waves for proper hand movement [15]. In 2012, Avanzini et al. reported the important role of alpha and beta brainwaves in motor activity [16]. They confirmed a suppression after each motor event, followed by a rebound of alpha and beta power at the sensorimotor cortex. The sensorimotor cortices brainwave data in this study, with 'on' and 'off' STN-DBS stimulation, disclosed nearly similar features to the previous findings. There was a marked reduction in the late theta and alpha wave power when the stimulation was 'on', and the patients showed clinical improvement. The Mu power wave changes signify the importance of Mu waves (theta and alpha) in producing optimal hand movement. In congruence with other studies, a gross morphological change in wave power was also noted for the beta waves when the STN was stimulated with clinical improvement [8-10]. However, a change in delta wave power was noted as minor, with a slight increment in wave power when STN was stimulated with clinical improvement.

Pertaining to the brainwave pattern, an interesting change to the pattern was observed when Parkinson’s patients underwent STN-DBS stimulation with clinical contralateral limb response. The asynchronized and incoherent brainwaves at motor cortices during the off-stimulation pattern soon change to a more synchronized and coherent pattern when the stimulation is ‘on’. Nonetheless, the obtained patterns during the 'on' state were not completely similar to healthy subject brainwaves, which have a distinctive double peaks feature at delta and alpha bands [17]. Therefore, STN-DBS also seems to enhance the synchronization of theta, alpha, and beta waves, giving rise to better brain function. This finding gives further literature support that the brain is a well-synchronized system with regard to its two hemispheres [18-20]. In conclusion, our findings highlight crucial evidence of optimal brain rhythms associated with movement execution for device development in brain-machine interfaces.

## Conclusions

STN-DBS works in alleviating the symptoms in Parkinson’s patients through alterations in brainwave oscillations. At the sensorimotor cortical level, the effect of STN stimulation seems to affect the brainwave power or energy for the Mu and beta waves and yields marked enhancement in wave synchrony and coherence at both sensorimotor cortices.

## References

[1] Błaszczyk JW (2017). Nigrostriatal interaction in the aging brain: new therapeutic target for Parkinson's disease. Acta Neurobiol Exp (Wars).

[2] Fu Y, Paxinos G, Watson C, Halliday GM (2016). The substantia nigra and ventral tegmental dopaminergic neurons from development to degeneration. J Chem Neuroanat.

[3] Braak H, Del Tredici K, Rub U, de Vos RAI, Jansen Steur ENH, Braak E (2003). Staging of brain pathology related to sporadic Parkinson's disease. Neurobiol Aging.

[4] Hilton D, Stephens M, Kirk L (2014). Accumulation of α-synuclein in the bowel of patients in the pre-clinical phase of Parkinson's disease. Acta Neuropathol.

[5] Liu B, Fang F, Pedersen NL (2017). Vagotomy and Parkinson disease. A Swedish register-based matched-cohort study. Neurology.

[6] Beudel M, Sadnicka A, Edwards M, de Jong BM (2019). Linking pathological oscillations with altered temporal processing in Parkinsons disease: neurophysiological mechanisms and implications for neuromodulation. Front Neurol.

[7] Timmermann L, Florin E (2012). Parkinson's disease and pathological oscillatory activity: is the beta band the bad guy? - New lessons learned from low-frequency deep brain stimulation. Exp Neurol.

[8] Yousif N, Bain PG, Nandi D, Borisyuk R (2020). A population model of deep brain stimulation in movement disorders from circuits to cells. Front Hum Neurosci.

[9] Hammond C, Bergman H, Brown P (2007). Pathological synchronization in Parkinson's disease: networks, models and treatments. Trends Neurosci.

[10] Zhao XM, Zhuang P, Li YJ, Zhang YQ, Li JY, Wang YP, Li JP (2020). Asymmetry of subthalamic neuronal firing rate and oscillatory characteristics in Parkinson's disease. Neuropsychiatr Dis Treat.

[11] Lachenmayer ML, Mürset M, Antih N (2021). Subthalamic and pallidal deep brain stimulation for Parkinson's disease-meta-analysis of outcomes. NPJ Parkinsons Dis.

[12] Chen CC, Hsu YT, Chan HL (2010). Complexity of subthalamic 13-35 Hz oscillatory activity directly correlates with clinical impairment in patients with Parkinson's disease. Exp Neurol.

[13] Kühn AA, Kupsch A, Schneider GH, Brown P (2006). Reduction in subthalamic 8-35 Hz oscillatory activity correlates with clinical improvement in Parkinson's disease. Eur J Neurosci.

[14] Weinberger M, Mahant N, Hutchison WD (2006). Beta oscillatory activity in the subthalamic nucleus and its relation to dopaminergic response in Parkinson's disease. J Neurophysiol.

[15] Ketenci S, Kayikcioglu T (2019). Investigation of theta rhythm effect in detection of finger movement. J Exp Neurosci.

[16] Avanzini P, Fabbri-Destro M, Dalla Volta R, Daprati E, Rizzolatti G, Cantalupo G (2012). The dynamics of sensorimotor cortical oscillations during the observation of hand movements: an EEG study. PLoS One.

[17] Idris Z, Zakaria Z, Reza F, Izaini Ghani AR, Abdullah JM (2022). Neuroscience and quantum physics aspect of human brainwaves. Multidisciplinarity and Interdisciplinarity in Health.

[18] Metzen MG, Hofmann V, Chacron MJ (2020). Neural synchrony gives rise to amplitude- and duration-invariant encoding consistent with perception of natural communication stimuli. Front Neurosci.

[19] Palacios ER, Isomura T, Parr T, Friston K (2019). The emergence of synchrony in networks of mutually inferring neurons. Sci Rep.

[20] Wang D, Huang Z, Ren L (2020). Amygdalar and hippocampal beta rhythm synchrony during human fear memory retrieval. Acta Neurochir (Wien).

